# Supplementary material for: Comprehensive analysis of molecular characteristic and clinical prognosis of CD8+ T cell related genes in idiopathic pulmonary fibrosis
Source: PLoS One. 2025 Jul 31;20(7):e0328250. doi: 10.1371/journal.pone.0328250 (PMC12312977; doi:10.1371/journal.pone.0328250)
Supplement: S1 File — (PDF) [file pone.0328250.s003.pdf]

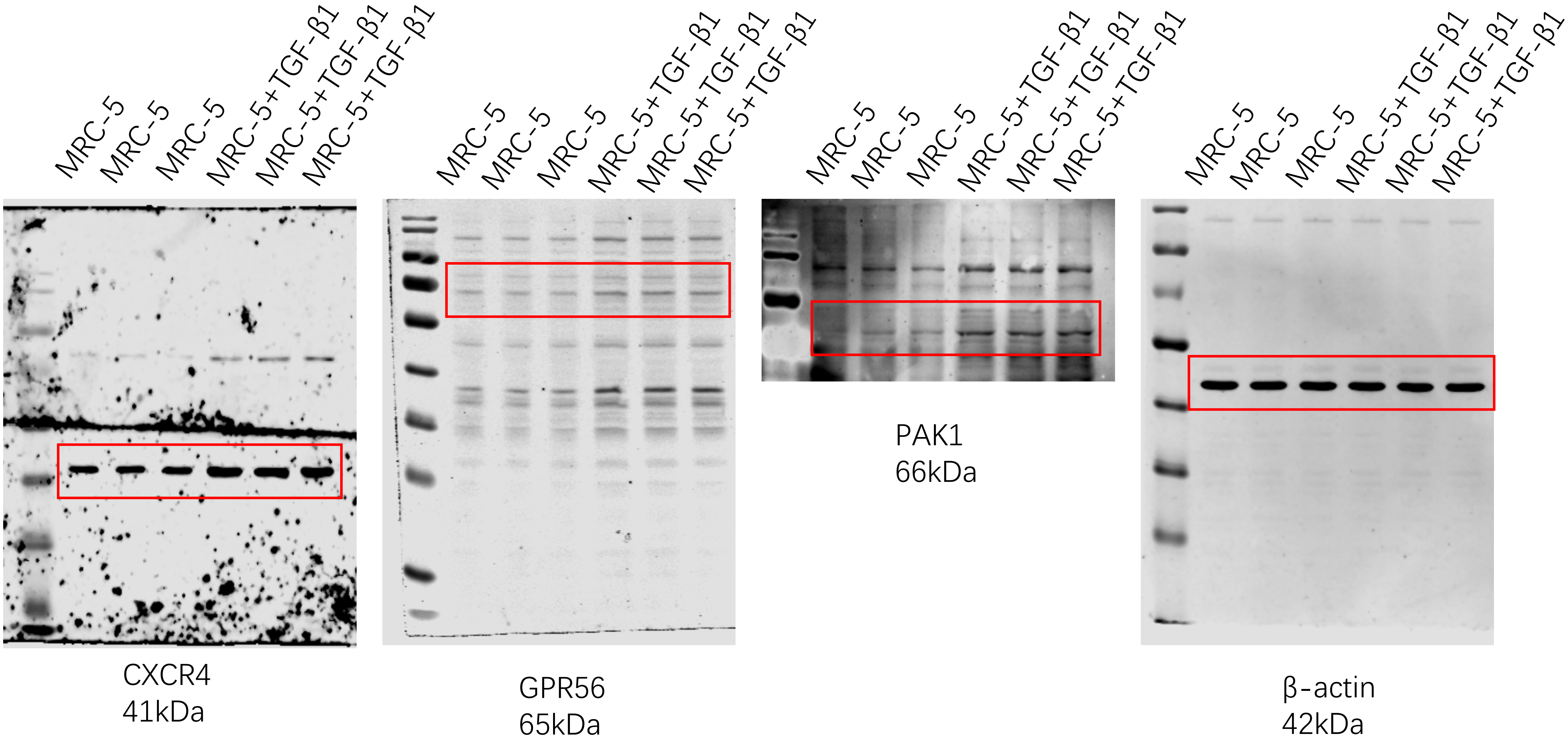
MRC-5
MRC-5
MRC-5
MRC-5+TGF-β1
MRC-5+TGF-β1
MRC-5+TGF-β1
CXCR4
41kDa
GPR56
65kDa
PAK1
66kDa
β-actin
42kDa
