# Supplementary material for: Comprehensive analysis of molecular characteristic and clinical prognosis of CD8+ T cell related genes in idiopathic pulmonary fibrosis
Source: PLoS One. 2025 Jul 31;20(7):e0328250. doi: 10.1371/journal.pone.0328250 (PMC12312977; doi:10.1371/journal.pone.0328250)
Supplement: Attachment — Submitted filename: Review_PONE-D-25-16281.docx [file pone.0328250.s004.docx]

**PONE-D-25-16281**

**Title of the manuscript:** *Comprehensive analysis of molecular characteristic and clinical prognosis of CD8+ T cell-associated genes in Idiopathic Pulmonary Fibrosis*

1. **Summary of the manuscript:**

The present study explores the role of CD8+ T cells in idiopathic pulmonary fibrosis (IPF) by identifying CD8+ T cell-associated genes associated with the prognosis (mainly survival) of the disease. Using transcriptomic data from the GSE70866 dataset, the authors apply WGCNA, differential expression analysis, and Cox regression to identify key prognostic markers – focusing on CXCR4, GRP56, and PAK1. These markers are used to construct a prognostic model and molecular subtypes of IPF. The model is validated using survival analysis and ROC curves. In addition, the results *in-silico* were validated *in vitro* using a TGF-β1-induced IPF cellular model, which confirmed the altered expression of the identified genes at both mRNA and protein levels. All in all, the study aims to provide new insights into IPF pathogenesis and potential prognostic biomarkers.

2. **General comments**

This manuscript addresses a clinically important topic and combines different bioinformatics approaches with experimental validation, which strengthens its translational relevance. The use of multiple statistical and machine learning techniques, such as WGCNA, LASSO, Cox regression, consensus clustering) is appropriate and well-aligned with the study goals. The paper is well written, the data is well presented and analyzed. However, the following general points should be addressed.

- The rationale for selecting CD8+ TCRGs and the source of the gene list is not clearly described.
- The manuscript would benefit from a clearer explanation of how the prognostic model was validated, particularly regarding the use of training and validation sets.
- The *in vitro* validation is a strong point, but more detail is needed on experimental replicates, controls, and statistical analysis.
- The manuscript should better clarify whether the identified markers are novel in the context of IPF or confirmatory of previous findings.

3. **Specific comments**
   3.1. **Major comments:**

Clarification of type of samples used: The manuscript does not clearly specify that the transcriptomic data from the GSE70866 dataset comes from bronchoalveolar lavage (BAL) cells. This is a critical detail that should be explicitly stated in the material and methods (IPF data set collection, line 77) and discussed in the context of the findings, as it influences the interpretation of immune-related gene expression and the relevance to lung tissue pathology in IPF.

Sample matching (IPF and controls): The manuscript describes the use of SVA and limma for batch correction and normalization, which is appropriate. However, it is unclear whether any matching or adjustment was performed to account for potential confounding variables, such as age or sex, between IPF and control groups. The authors should clarify whether such matching was considered or whether the groups were balanced.

Interpretation of WGCNA results: The authors identify and focus on the cyan module as the most correlated with CD8+ T cells (r = 0.85, p = 2e-56). However, the biological interpretation of this module is not discussed. What types of genes are enriched in this module? Are there known pathways or functions?

Molecular Subtyping: The authors identify two molecular subtypes (A and B) based on CD8+ TCRGs expression, with subtype A associated with worse clinical prognosis survival and higher immune infiltration. While the clustering appears statistically sound, the biological implications of these subtypes are not fully explored.

Training and validation sets: The authors mentioned splitting the dataset in two (training and validation set), but it is unclear whether this was done for the IPF dataset or mistakenly refers to a "sepsis" dataset (line 117). Please clarify and ensure consistency in terminology.

Sankey Diagram: The Sankey diagram (Figure 4G) is visually informative, but the manuscript does not explain its significance. What new information does the flow between subtypes, risk groups, and outcomes reveal about the model’s utility?

Prognostic analysis: In the prognostic analysis of CD8+ TCRGs score, the manuscript includes only age, gender, and GAP index as “clinical pathological features”. As a composite, GAP may mask the individual contribution of the variables that it includes, and also does not account for disease progression. That is why, since the study focuses on immune gene expression, it would be interesting to incorporate individual parameters: lung function and diffusion capacity (FVC, DLCO), 6-minute walk distance, exacerbations or comorbidities, rather than the GAP score. This might provide more insights into the relationship between molecular subtypes and clinical outcomes and enhance the prognostic model. However, as these data are not available in the GSE70866 dataset, this limits the ability to fully assess the clinical correlation and independence of the proposed molecular risk model. The authors should acknowledge this limitation and discuss its implications.

Link Between BAL findings and the MRC-5 in vitro validation: The manuscript identifies prognostic genes from transcriptomic data derived from BAL cells, which primarily reflect immune cell activity. However, the experimental validation is conducted in MRC-5 fibroblasts, which are mesenchymal cells. The authors should more clearly explain the biological rationale connecting these two systems. Specifically: how do the gene expression changes observed in immune cells relate to those induced in fibroblasts by TGF-β1? A discussion of shared signaling pathways or cross-talk between immune and fibroblasts in IPF would help consolidate the interpretation of the findings.

Limitations and future research directions: The authors acknowledge several limitations, including the lack of in-depth mechanistic studies and the use of cell line data. These are valid points, however, the discussion could be strengthened by also suggesting specific future research directions to address the mentioned gaps.

3.2. **Minor comments:**

Suggestion- including literature on blood immune profiles: The introduction could benefit from referencing recent studies that explore systemic immune signatures in IPF. For example, the study by Mendoza et al (<https://pubmed.ncbi.nlm.nih.gov/37762135/>) investigates peripheral blood immune profiles and their association with IPF progression (based on lung function decline and mortality). This could help contextualize the current study within the broader landscape of immune-based prognostic research in IPF.

Line 79 – I recommend using the term “healthy donors” or “healthy controls”, instead of “normal samples”-

Line 84 – Specify which are the “other clinical pathological parameters”.

The authors should ensure that all abbreviations are clearly defined at first use (WGCNA, etc).

The abbreviation “CD8+ TCRGs” can be unclear and potentially misleading, as it may be interpreted as “CD8+ T Cell Receptor Genes.” If the authors are referring to genes associated with CD8+ T cell activity rather than TCR genes specifically, a different term or abbreviation should be used to avoid confusion. Maybe CD8+ TRGs (T cell Related Genes) or TAGs (T cell Associated genes).

Line 236: “GPR56” should be “GRP56”, if consistent with earlier usage.

Line 265 to 268 – There is a figure legend misplaced here.

The figures in the manuscript appear to be of low resolution, making it difficult to interpret key visual elements. The authors should ensure that all figures are provided in high resolution and that all text and symbols are clearly legible.

Figure 8 (A-D) labels are a bit confusing. I would propose to use the terms MRC-5 and MRC-5+TGFbeta or similar, so it is clearer which one is treated with TGFbeta.

Ensure that all abbreviations in figures and tables are defined in the legends.

4. **Recommendation**

Based on all the above, I believe that this manuscript is **suitable for publication after major revision**. It may not be groundbreaking, but it contributes meaningfully to the field and opens for further research.
