# Supplementary material for: Comprehensive analysis of molecular characteristic and clinical prognosis of CD8+ T cell related genes in idiopathic pulmonary fibrosis
Source: PLoS One. 2025 Jul 31;20(7):e0328250. doi: 10.1371/journal.pone.0328250 (PMC12312977; doi:10.1371/journal.pone.0328250)
Supplement: Attachment — Submitted filename: Response to Reviewers.docx [file pone.0328250.s006.docx]

We sincerely thank the editor and reviewers for their constructive comments and suggestions. We have carefully revised our manuscript accordingly. Below are our point-by-point responses to each comment.

**PONE-D-25-16281**

**Title of the manuscript:** *Comprehensive analysis of molecular characteristic and clinical prognosis of CD8+ T cell-associated genes in Idiopathic Pulmonary Fibrosis*

**1: Summary of the manuscript:**

The present study explores the role of CD8+ T cells in idiopathic pulmonary fibrosis (IPF) by identifying CD8+ T cell-associated genes associated with the prognosis (mainly survival) of the disease. Using transcriptomic data from the GSE70866 dataset, the authors apply WGCNA, differential expression analysis, and Cox regression to identify key prognostic markers – focusing on CXCR4, GRP56, and PAK1. These markers are used to construct a prognostic model and molecular subtypes of IPF. The model is validated using survival analysis and ROC curves. In addition, the results *in-silico* were validated *in vitro* using a TGF-β1-induced IPF cellular model, which confirmed the altered expression of the identified genes at both mRNA and protein levels. All in all, the study aims to provide new insights into IPF pathogenesis and potential prognostic biomarkers.

2: **General comments**

This manuscript addresses a clinically important topic and combines different bioinformatics approaches with experimental validation, which strengthens its translational relevance. The use of multiple statistical and machine learning techniques, such as WGCNA, LASSO, Cox regression, consensus clustering) is appropriate and well-aligned with the study goals. The paper is well written, the data is well presented and analyzed. However, the following general points should be addressed.

1: The rationale for selecting CD8+ TCRGs and the source of the gene list is not clearly described.

Reply：

Thank you very much for this insightful and critical comment. Previous studies have demonstrated that alterations in the immune microenvironment play a crucial role in the onset and progression of IPF. Therefore, a central aim of our study was to systematically investigate the potential associations between the immune microenvironment composition and IPF.

To begin with, we employed the ssGSEA algorithm to quantitatively analyze the infiltration levels of 23 immune cell types, aiming to identify those most closely associated with IPF. Subsequently, we utilized the WGCNA algorithm to assess co-expression relationships between these immune cell types and the IPF expression profile. Our results revealed that CD8+ T cells exhibited the strongest correlation with IPF, suggesting that they may represent a key immune component involved in the progression of the disease.

Building on this finding, we extracted a core gene signature from the WGCNA module most strongly associated with CD8+ T cells and conducted further in-depth analyses of these genes. In the subsequent steps, we evaluated the prognostic relevance of these genes in IPF patients and developed a CD8+ T cell-related gene (CRG) signature-based prognostic model, referred to as the CRG score, to stratify patient risk.

Once again, we sincerely appreciate the reviewer for pointing out the lack of clarity in this section. In the revised manuscript, we have added and clarified detailed information regarding the genes included in this module to enhance the logical coherence and readability of the study.

**Supplementary table 2, Line 104**

2: The manuscript would benefit from a clearer explanation of how the prognostic model was validated, particularly regarding the use of training and validation sets.

Reply：

We sincerely thank the reviewer for the valuable comment. We fully agree with your suggestion and have revised the manuscript to provide a more detailed and clearer description of the construction and validation process of the prognostic model.

In this study, we used the “caret” R package to randomly divide all IPF patient samples into a training set and a validation set at a 7:3 ratio. The training set was utilized to perform univariate Cox regression, LASSO regression, and multivariate Cox regression analyses to construct the prognostic risk score model. The validation set was then used to evaluate the robustness and predictive performance of the constructed model.

In both independent cohorts, we generated Kaplan-Meier survival curves and time-dependent ROC curves, and compared their AUC values to assess the model’s consistency and accuracy across datasets.

We believe that these additions help to improve the overall clarity and persuasiveness of the manuscript. Once again, we are grateful for your insightful suggestions.

**Line 107-125**

3: The *in vitro* validation is a strong point, but more detail is needed on experimental replicates, controls, and statistical analysis.

Reply：

We sincerely thank the reviewer for the positive evaluation of our in vitro experiments and for the thoughtful and constructive suggestions for improvement. In the revised manuscript, we have provided more detailed descriptions in the Methods section, as outlined below:

1. **Experimental reproducibility**: All in vitro experiments were independently repeated at least three times to ensure the reliability and reproducibility of the results.
2. **Control settings**: In our cell experiments, we established clear control (MRC-5) and model (MRC-5 + TGF-β1) groups to evaluate the changes in mRNA and protein expression levels of the target gene under different conditions.
3. **Statistical analysis**: All statistical analyses were performed using GraphPad Prism 8.0. Two-tailed Student’s *t*-tests were used for comparisons between two groups, and significance levels (*P* < 0.05, *P* < 0.01, *P* < 0.001) were clearly indicated in the figure legends. Data were presented as mean ± standard deviation (SD).

We believe that these additions help to improve the rigor and scientific credibility of the in vitro experimental section. Once again, we are grateful to the reviewer for their careful evaluation and valuable feedback.

**Line 172-183, line 329-342, Figure 8**

4: The manuscript should better clarify whether the identified markers are novel in the context of IPF or confirmatory of previous findings.

Reply：

We sincerely thank the reviewer for the valuable suggestions provided for our study. In response to your comments, we have expanded the *Discussion* section to include a more comprehensive interpretation of the key biomarkers identified in the context of IPF, and further elaborated on the novelty of our findings.

Specifically, we conducted a systematic literature review and comparative analysis of the previously reported roles of the selected genes in IPF-related studies. Our results indicate that **PAK1** and **GPR56** have not been previously reported to be associated with IPF, making our study the first to propose their potential involvement in the pathogenesis of the disease. This highlights the novelty and exploratory value of our findings. In contrast, **CXCR4** has been documented in prior studies, which confirmed its upregulation in lung tissues of IPF patients and suggested its potential role in disease progression and in predicting responses to pirfenidone treatment (Relevant references: PMID: 29507348, 32843095, 32822674, 29453386). Our results further validate and support the functional relevance of CXCR4 in IPF.

We believe that these additions enhance the logical flow and comprehensiveness of the *Discussion* section, while also better emphasizing the academic value and significance of our study. Once again, we thank the reviewer for their attention to and guidance on our work.

**Line 381-3894, Line 391-393**

**3：Specific comments**

**Major comments:**

1: Clarification of type of samples used: The manuscript does not clearly specify that the transcriptomic data from the GSE70866 dataset comes from bronchoalveolar lavage (BAL) cells. This is a critical detail that should be explicitly stated in the material and methods (IPF data set collection, line 77) and discussed in the context of the findings, as it influences the interpretation of immune-related gene expression and the relevance to lung tissue pathology in IPF.

Reply：

We sincerely appreciate the reviewer’s professional and important suggestion. In response to this concern, we have explicitly clarified the source of the GSE70866 dataset in the *Materials and Methods* section, stating that it comprises transcriptomic data derived from bronchoalveolar lavage (BAL) cells of patients with IPF.

Additionally, we have expanded the *Discussion* section to further explain the rationale for using BAL cells as samples for immune profiling. We have briefly discussed both their representativeness and limitations in elucidating the immunopathological mechanisms of IPF, aiming to help readers better understand the study context and interpret the results more accurately.

We believe that these revisions enhance the scientific rigor and readability of the manuscript. Once again, we thank the reviewer for their thorough evaluation and constructive feedback.

**Line 351-366**

2: Sample matching (IPF and controls): The manuscript describes the use of SVA and limma for batch correction and normalization, which is appropriate. However, it is unclear whether any matching or adjustment was performed to account for potential confounding variables, such as age or sex, between IPF and control groups. The authors should clarify whether such matching was considered or whether the groups were balanced.

Reply:

We sincerely thank the reviewer for raising this critical and insightful question. We fully agree that controlling for potential confounding factors such as age and sex is essential to ensure the reliability and scientific validity of group comparison analyses.

In this study, prior to data integration and downstream analyses, we carefully reviewed the clinical information of the included datasets, with particular attention to the distribution of age and sex between the IPF and normal control groups. It is important to note that although the two platform files were generated from different sources, they both belong to the same GEO dataset and include complete clinical feature information for all samples. Preliminary assessments indicated that the distribution of age and sex was relatively balanced between groups.

To further minimize potential bias from platform batch effects and confounding variables, we applied standardization and batch effect correction using the **SVA** package. These methodological details have been added to the revised manuscript. Additionally, we have included baseline clinical characteristics of the samples in the *Materials and Methods* section and provided them as **Supplementary Table 1**, to improve the transparency and reproducibility of our data processing.

We believe these revisions strengthen the rigor and persuasiveness of our study. Once again, we sincerely thank the reviewer for the thoughtful review and valuable suggestions.

**Supplementary table 1, line 83-line 93**

3： Interpretation of WGCNA results: The authors identify and focus on the cyan module as the most correlated with CD8+ T cells (r = 0.85, p = 2e-56). However, the biological interpretation of this module is not discussed. What types of genes are enriched in this module? Are there known pathways or functions?

Reply:

We sincerely thank the reviewer for the valuable comments. In response to your suggestion, we have conducted a comprehensive functional enrichment analysis of the genes within the cyan module to further explore their potential biological significance.

Specifically, we performed Gene Ontology (GO) and Kyoto Encyclopedia of Genes and Genomes (KEGG) enrichment analyses. The results revealed that the genes in this module are significantly enriched in various immune-related biological processes and signaling pathways, including the T cell receptor signaling pathway, T cell activation, and chemokine signaling pathway. These findings suggest that the cyan module may play a critical role in CD8⁺ T cell-mediated immune responses, thereby further supporting the scientific rationale and validity of using this module as the basis for constructing our prognostic model.

These functional annotations have been added to the revised manuscript in the Results section. Once again, we sincerely thank the reviewer for the professional suggestion, which has substantially enhanced the depth and rigor of our study.

**Figure 1E, F, line 196-205**

4：Molecular Subtyping: The authors identify two molecular subtypes (A and B) based on CD8+ TCRGs expression, with subtype A associated with worse clinical prognosis survival and higher immune infiltration. While the clustering appears statistically sound, the biological implications of these subtypes are not fully explored.

Reply：

We sincerely appreciate the reviewer’s interest in our molecular subtype analysis and the constructive suggestions provided. In response to your comments, we have conducted a more in-depth and systematic comparative analysis of the biological characteristics of subtype A and subtype B during the revision process.

Specifically, we evaluated the differences between the two subtypes in terms of key signaling pathway enrichment and immune cell infiltration levels. The results revealed that subtype A not only exhibited a poorer survival outcome but also showed significant enrichment of its signature genes in several canonical immune-related pathways, such as leukocyte migration, chemotaxis, immune receptor activity, cytokine–cytokine receptor interaction, and chemokine signaling pathway. These findings suggest that subtype A may be in a state of “immune activation accompanied by immune exhaustion,” a feature that has also been observed in certain autoimmune diseases and tumor immune microenvironments.

Furthermore, we employed the single-sample Gene Set Enrichment Analysis (ssGSEA) method to assess the distribution of various immune cell types across the two subtypes. The results demonstrated that subtype A had significantly higher infiltration levels of CD8⁺ T cells, B cells, and CD4⁺ T cells, further supporting the hypothesis of an immune-activated state in subtype A.

These results not only help to explain the potential immunological mechanisms underlying the prognostic differences between the molecular subtypes but also provide a theoretical basis for the future development of precision therapeutic strategies. We have incorporated the relevant findings into the Discussion section of the revised manuscript. Once again, we sincerely thank the reviewer for the insightful suggestions, which have greatly contributed to the improvement of our study.

**Line 351-366**

5：Training and validation sets: The authors mentioned splitting the dataset in two (training and validation set), but it is unclear whether this was done for the IPF dataset or mistakenly refers to a "sepsis" dataset (line 117). Please clarify and ensure consistency in terminology.

Reply：

We sincerely thank the reviewer for pointing out the ambiguity in our dataset partitioning and terminology usage. In response to this issue, we have conducted a thorough review and correction of the relevant content in the revised manuscript.

Upon careful verification, we confirmed that the original use of the term “sepsis dataset” was incorrect; the accurate term should be “IPF dataset.” Specifically, we randomly divided 176 IPF patient samples with complete clinical information from the GSE70866 dataset into a training set (n = 124) and a validation set (n = 52) at a ratio of 7:3. The training set was used for constructing the prognostic model, including univariate Cox regression, LASSO regression, and multivariate Cox regression analyses. The validation set was used to assess and verify the model’s performance, thereby ensuring its robustness and generalizability.

We have updated and standardized the relevant terminology in the “Materials and Methods” section and added a detailed description of the sample partitioning criteria and workflow. These revisions ensure consistency, accuracy, and improve the reproducibility and clarity of our study. Once again, we sincerely appreciate the reviewer’s careful review and valuable suggestions.

**Line 107-125**

6: Sankey Diagram: The Sankey diagram (Figure 4G) is visually informative, but the manuscript does not explain its significance. What new information does the flow between subtypes, risk groups, and outcomes reveal about the model’s utility?

Reply:

We sincerely thank the reviewer for the insightful comments and suggestions regarding the Sankey diagram presented in Figure 4G. We acknowledge that the original manuscript did not provide sufficient explanation of the scientific and clinical implications of this figure. In the revised manuscript, we have added a detailed interpretation.

The Sankey diagram visually illustrates the relationships among CRG molecular subtypes, risk score groups, and patient prognostic outcomes (survival status). The figure clearly demonstrates that:

(1) The majority of patients classified as subtype A are assigned to the high-risk group and tend to have poorer survival outcomes;

(2) In contrast, patients with subtype B are predominantly distributed in the low-risk group and exhibit better survival status;

(3) The risk score serves as an effective bridge between molecular subtypes and clinical prognosis, further validating the model’s ability to stratify survival risk among patients with distinct immune subtypes.

This figure not only highlights the close association between subtype classification and risk stratification but also suggests the potential clinical utility of the model in guiding prognostic assessment and personalized treatment strategies. We have reinforced the interpretation of Figure 4G in the Results section and expanded the discussion of its clinical significance.

**Line 266-276**

7：Prognostic analysis: In the prognostic analysis of CD8+ TCRGs score, the manuscript includes only age, gender, and GAP index as “clinical pathological features”. As a composite, GAP may mask the individual contribution of the variables that it includes, and also does not account for disease progression. That is why, since the study focuses on immune gene expression, it would be interesting to incorporate individual parameters: lung function and diffusion capacity (FVC, DLCO), 6-minute walk distance, exacerbations or comorbidities, rather than the GAP score. This might provide more insights into the relationship between molecular subtypes and clinical outcomes and enhance the prognostic model. However, as these data are not available in the GSE70866 dataset, this limits the ability to fully assess the clinical correlation and independence of the proposed molecular risk model. The authors should acknowledge this limitation and discuss its implications.

Reply：

We sincerely thank the reviewer for the valuable suggestions. We fully agree that while composite indices such as the GAP score are convenient for overall assessment, they may obscure the individual contributions of key clinical parameters and fail to dynamically reflect disease progression.
In light of this, we have added new content in the Discussion section to elaborate on the potential significance and future application prospects of incorporating individual clinical variables—such as pulmonary function measures (e.g., FVC and DLCO) and the six-minute walk distance—into future studies.
Unfortunately, due to the lack of such detailed clinical data in the GSE70866 dataset, we were unable to perform further in-depth clinical correlation analyses in the current study. We have clearly stated and discussed this limitation in the revised manuscript.
Once again, we sincerely appreciate the reviewer’s constructive comments, which have greatly contributed to enhancing the depth and rigor of our study.

**Line 423-445**

8：Link Between BAL findings and the MRC-5 in vitro validation: The manuscript identifies prognostic genes from transcriptomic data derived from BAL cells, which primarily reflect immune cell activity. However, the experimental validation is conducted in MRC-5 fibroblasts, which are mesenchymal cells. The authors should more clearly explain the biological rationale connecting these two systems. Specifically: how do the gene expression changes observed in immune cells relate to those induced in fibroblasts by TGF-β1? A discussion of shared signaling pathways or cross-talk between immune and fibroblasts in IPF would help consolidate the interpretation of the findings.

Reply：

We thank the reviewer for the insightful comments regarding the biological rationale linking BAL-derived transcriptomic data to the MRC-5 in vitro validation model. We agree that, while this discrepancy may initially appear to be a limitation, it in fact underscores the complex immune–mesenchymal interactions underlying IPF pathogenesis. To further clarify this connection, we have revised the Discussion section accordingly.
We now explicitly state that the gene expression alterations observed in immune cells may reflect dynamic crosstalk between immune cells and fibroblasts, potentially involving shared activation pathways that ultimately influence fibroblast phenotypes. We suggest that future studies should aim to establish co-culture systems of immune cells and fibroblasts to more directly assess their functional interactions and to validate the mechanistic relevance of the identified prognostic genes.
We believe these revisions help to strengthen the biological plausibility of our findings and directly address the reviewer’s concerns. Moreover, the use of MRC-5 cells to validate findings from BAL-derived data is a commonly adopted approach, as supported by previously published studies (PMIDs: 39872532, 30021376, 20388759, 24890593).
Once again, we sincerely thank the reviewer for the constructive feedback, which has helped us to improve the quality and clarity of our manuscript.

**Line 351-366**

9：Limitations and future research directions: The authors acknowledge several limitations, including the lack of in-depth mechanistic studies and the use of cell line data. These are valid points, however, the discussion could be strengthened by also suggesting specific future research directions to address the mentioned gaps.

Reply：

Thank you for the constructive comments. We have revised the Discussion section to include several specific recommendations for future research. These include conducting in vivo studies to further investigate the mechanistic role of the CRG scoring system in immune regulation and disease progression, as well as applying single-cell sequencing and spatial transcriptomics to better characterize the heterogeneity of the tumor immune microenvironment.
Additionally, we recommend that future prospective clinical studies incorporate comprehensive patient-level data to validate and refine the prognostic utility of our molecular risk model.
We believe that these additions will help to build upon our current findings and lay the foundation for more robust investigations in the future.

**Line 423-445**

**4：Minor comments:**

1：Suggestion- including literature on blood immune profiles: The introduction could benefit from referencing recent studies that explore systemic immune signatures in IPF. For example, the study by Mendoza et al (<https://pubmed.ncbi.nlm.nih.gov/37762135/>) investigates peripheral blood immune profiles and their association with IPF progression (based on lung function decline and mortality). This could help contextualize the current study within the broader landscape of immune-based prognostic research in IPF.

Reply:

We sincerely thank the reviewer for the valuable suggestion. We fully agree that incorporating recent studies on systemic immune signatures into the Introduction can enhance the theoretical background and clinical relevance of our study.

Accordingly, we have added a summary of the study by Mendoza et al., published in 2023, to the Introduction section. We briefly described how this study revealed the association between peripheral blood immune profiles and IPF prognosis. This addition further supports the scientific rationale of our focus on immune-related prognostic markers and helps to better position our work within the context of existing research.

**Line 57-61**

2：Line 79 – I recommend using the term “healthy donors” or “healthy controls”, instead of “normal samples”-

Reply：

We sincerely thank the reviewer for the detailed suggestion. We agree that the terms “healthy donors” or “healthy controls” are more accurate and professional than “normal samples,” as they more clearly convey the source and characteristics of the control group.
Accordingly, we have revised the manuscript by replacing “normal samples” with “healthy donors” as suggested. We greatly appreciate the reviewer’s insightful comment.

3：Line 84 – Specify which are the “other clinical pathological parameters”.

Reply：

We sincerely thank the reviewer for pointing out the lack of specificity in this section. To provide a clearer description, we have supplemented the manuscript with detailed clinical and pathological parameters, including age, sex, and GAP index. These details have been thoroughly described in the revised manuscript to facilitate a better understanding of the clinical characteristics involved.

We appreciate the reviewer’s attention to this important detail.

**Line 91-93, Supplementary table 1**

4：The authors should ensure that all abbreviations are clearly defined at first use (WGCNA, etc).

Reply：

We sincerely thank the reviewer for the careful reminder. We have thoroughly reviewed the entire manuscript to ensure that all abbreviations, including key terms such as WGCNA, are fully and clearly defined upon their first appearance. These revisions have been made to improve the manuscript’s standardization and readability.

5：The abbreviation “CD8+ TCRGs” can be unclear and potentially misleading, as it may be interpreted as “CD8+ T Cell Receptor Genes.” If the authors are referring to genes associated with CD8+ T cell activity rather than TCR genes specifically, a different term or abbreviation should be used to avoid confusion. Maybe CD8+ TRGs (T cell Related Genes) or TAGs (T cell Associated genes).

Reply：

We sincerely thank the reviewer for the important suggestion. We agree that the abbreviation “CD8⁺ TCRGs” may cause misunderstanding; therefore, we have changed it to “CRG” to more accurately represent the relevant genes. Corresponding expressions throughout the manuscript have also been updated accordingly to avoid ambiguity. We greatly appreciate the reviewer’s careful guidance, which has helped us improve the professionalism and clarity of our manuscript.

6：Line 236: “GPR56” should be “GRP56”, if consistent with earlier usage.

Reply：

Thank you for pointing out the spelling issue. We have reviewed the original data and confirmed that the correct spelling is GPR56 (G Protein-Coupled Receptor 56). We have carefully checked the entire manuscript and made the necessary corrections accordingly.

7：Line 265 to 268 – There is a figure legend misplaced here.

Reply:

We thank the reviewer for pointing out the formatting error introduced during the editing process. In the revised manuscript, we have removed the redundant figure legend accordingly.

8：The figures in the manuscript appear to be of low resolution, making it difficult to interpret key visual elements. The authors should ensure that all figures are provided in high resolution and that all text and symbols are clearly legible.

Reply：

We thank the reviewer for highlighting the issue of image resolution. We have reprocessed and replaced all figures to ensure they are in high-resolution format, with clear and legible text and symbols. We hope these improvements enhance the presentation quality of the figures, facilitating better understanding and analysis by readers.

9：Figure 8 (A-D) labels are a bit confusing. I would propose to use the terms MRC-5 and MRC-5+TGFbeta or similar, so it is clearer which one is treated with TGFbeta.

Reply：

We sincerely thank the reviewer for the valuable suggestion. We agree that the labels in Figure 8 were not sufficiently intuitive. To enhance clarity, we have revised the labels to “MRC-5” and “MRC-5 + TGFβ” to clearly distinguish the treatment group from the control group. These changes have been made accordingly in Figure 8.

10：Ensure that all abbreviations in figures and tables are defined in the legends.

Reply:

We sincerely thank the reviewer for the careful reminder. We have thoroughly checked all figures and their legends to ensure that all abbreviations are clearly explained for readers’ better understanding. The relevant revisions have been completed.

1. **Recommendation**

Based on all the above, I believe that this manuscript is **suitable for publication after major revision**. It may not be groundbreaking, but it contributes meaningfully to the field and opens for further research.
