# Supplementary material for: Comprehensive analysis of molecular characteristic and clinical prognosis of CD8+ T cell related genes in idiopathic pulmonary fibrosis
Source: PLoS One. 2025 Jul 31;20(7):e0328250. doi: 10.1371/journal.pone.0328250 (PMC12312977; doi:10.1371/journal.pone.0328250)
Supplement: Attachment — Submitted filename: Response_to_Reviewers_auresp_2.docx [file pone.0328250.s007.docx]

Reviewer #2: Thank you for the opportunity to review this well conducted and very interesting study that further supports the Role of immunity in IPF. This study was an unmet need. While new data were available for monocytes and Tregs in IPF, there was a need for such studies for CD8 T cells. The analysis is elegant and the figures are nice ( volcano plots, Survival curves, heat maps etc all of them are elegant). Some comments that could even improve more this manuscript are provided. They mainly reflect the effort to identify therapeutic targets and to further improve the discussion based on other recent works for immunity in IPF. Specific comments follow:

1：Authors should do connectivity map analysis using as input the CD8 related genes that they found to find drugs that increase or decrease these genes. This can help the field move on. For example they can use the Clue platform for that which accepts as input up to 250 genes ( in case the genes are more, those that have the higher log2fold change are selected).

Reply:

We sincerely thank the reviewer for this insightful and constructive suggestion. We fully agree that performing a Connectivity Map (CMap) analysis based on CD8-related genes may help identify potential compounds that modulate these targets and thereby provide valuable clues for developing immunotherapeutic strategies.

In fact, we attempted to conduct CMap analysis using the Clue platform during our exploratory analysis. However, the number of CD8-related differentially expressed genes identified in our study was relatively limited (only 48 genes), and the analysis did not yield statistically significant or biologically meaningful drug predictions. As a result, we decided not to include this part in the current manuscript in order to maintain analytical rigor.

Nevertheless, we have added a description of this exploratory attempt and its limitation in the revised Discussion section. We also pointed out that future studies, potentially based on expanded sample size or integrated immune-related signatures, could allow more robust drug prediction through CMap or similar approaches.

Once again, we appreciate your thoughtful suggestion, which has provided us with an important direction for future research.

2： Authors should substantially improve their discussion. First of all, it would be better to start it with the main findings of this work and not with introductory sentences. Secondly authors need to discuss more immune abberrations in IPF and how their work is relevant compared to other recent works. Single cell RNA sequencing studies in IPF found an outcome-predictive increase in classical monocytes and T regulatory cells. Further investigation showed that CD14+CD163–HLA-DRlow monocytes were the monocytes that were most consistently associated with progression. These monocytes seem to be associated with expansion of Tregs, suppression of natural killer cells and suppression of some T cell sub populations. This could be the reason that expansion of Tregs, and suppression of natural killer cells have been associated with IPF progression.

Further research aiming to address whether CD8 T cells are part of the same cascade and for their exact interaction with other immune subpopulations is greatly anticipated.

The studies that describe the above are provided here and should be in the reference list along with a more detailed presentation of immunity in IPF in the discussion.

https://pubmed.ncbi.nlm.nih.gov/38717443/

https://www.medrxiv.org/content/10.1101/2024.08.07.24311386v2

https://pmc.ncbi.nlm.nih.gov/articles/PMC8491266/

https://pmc.ncbi.nlm.nih.gov/articles/PMC12001815/

https://pmc.ncbi.nlm.nih.gov/articles/PMC3570653/

https://www.thelancet.com/journals/ebiom/article/PIIS2352-3964(23)00332-8/fulltext

https://pubmed.ncbi.nlm.nih.gov/39510135/

- <https://pubmed.ncbi.nlm.nih.gov/38717443/>
- <https://www.medrxiv.org/content/10.1101/2024.08.07.24311386v2>
- <https://pmc.ncbi.nlm.nih.gov/articles/PMC8491266/>
- <https://pmc.ncbi.nlm.nih.gov/articles/PMC12001815/>
- <https://pmc.ncbi.nlm.nih.gov/articles/PMC3570653/>
- <https://www.thelancet.com/journals/ebiom/article/PIIS2352-3964(23)00332-8/fulltext>
- <https://pubmed.ncbi.nlm.nih.gov/39510135/>

Reply：

We sincerely appreciate the reviewer’s insightful comments, which have helped us significantly improve the discussion section of our manuscript. As suggested, we have restructured the discussion to begin with a clear summary of our main findings before elaborating on their implications. Additionally, we have expanded our discussion on immune aberrations in IPF, particularly the role of Tregs, and their interactions with other immune subsets such as NK cells and effector T cells. We now highlight recent single-cell RNA sequencing studies linking CD14+CD163–HLA-DRlow monocytes to disease progression and their potential role in Treg expansion and NK cell suppression, aligning our findings with this emerging evidence. We have also incorporated the recommended references into the revised manuscript, which further strengthens the scientific rigor and context of our work. We are grateful for these suggestions, as they have enhanced the depth and accuracy of our discussion. Thank you for your valuable feedback.

**Line 369-Line 383**

3) Authors should change a title of the results by writing “Validation of the accuracy” instead of “Validate the accuracy of” so that they are consistent with the other titles of results

Reply：

We sincerely appreciate the reviewer’s careful attention to detail. As suggested, we have revised the title of the results section from "Validate the accuracy of" to "Validation of the accuracy" to maintain consistency with the other section titles. Thank you for this helpful correction.

**Line 277**
